# IS ACTIVE AGING POSSIBLE IN ETHIOPIA? PERCEPTIONS OF RURAL OLDER ADULTS

**DOI:** 10.1093/geroni/igac059.2435

**Published:** 2022-12-20

**Authors:** Abraham Teshome, Messay Kotecho, Margaret Adamek

**Affiliations:** Indiana University, Indianapolis, Indiana, United States; Addis Ababa University, Addis Ababa, Adis Abeba, Ethiopia; Indiana University, Indianapolis, Indiana, United States

## Abstract

Despite historical veneration of elders, older adults are increasingly viewed as a burden in many Sub Saharan African nations. Using a hermeneutic phenomenological approach, in-depth interviews were conducted to explore the aging experiences of 20 adults aged 70 and older in rural Ethiopia. Themes that emerged from the interviews were analyzed in light of the three pillars of the World Health Organization’s Active Aging Framework: health, security, and participation. Despite facing multiple barriers to active aging including lack of health care, financial hardship, ageism, and social exclusion, study participants were determined not to withdraw from activities in an effort to retain their autonomy, independence, and sense of dignity. As one participant shared, "the government does not care about us because it considers us as a useless segment of the society." Three themes reflected their major struggles: dwindling health and lack of access to health care, financial hardship, and social exclusion tied to ageism. The fourth theme--willful and purposive engagement--reflects the older adults’ response to these struggles. Contrary to myths about rural aging, study participants faced multifaceted challenges that kept them from realizing active ageing as they were not provided with sustained opportunities for health, security, and participation. Study findings point to the need for policymakers and other concerned bodies to develop supportive policies and programs to promote older adults’ well-being. The study calls for a paradigm shift that involves adopting the WHO’s Active Aging Framework, developing rights-based policies and programs, popularizing active aging, and revitalizing intergenerational solidarity.

